# Epidemiology of underweight and overweight-obesity among term pregnant Sudanese women

**DOI:** 10.1186/1756-0500-3-327

**Published:** 2010-12-06

**Authors:** Duria A Rayis, Ameer O Abbaker, Yasir Salih, Tayseer E Diab, Ishag Adam

**Affiliations:** 1 Faculty of Medicine University of Khartoum, Khartoum, Sudan; 2 Faculty of Medicine, Elneileen University, Khartoum, Sudan

## Abstract

**Background:**

The increasing prevalence of obesity in young women is a major public health concern. Few data are available concerning the epidemiology of malnutrition especially obesity among pregnant women in the developing countries. A cross sectional study was conducted at Khartoum hospital during February-April 2008, to investigate prevalence of underweight, obesity, and to identify contemporary socio-demographic predictors for obesity among term pregnant women in Khartoum Hospital, Sudan. After taking an informed consent, a structured questionnaire was administered to each woman to gather information on educational level, age and parity. Maternal weight and height were measured and expressed as body mass index (BMI - weight (kg)/height (m) ^2^).

**Findings:**

Out of 1690 term pregnant women, 628 (37.1%) were primigravidae, 926 (54.8%) had ≥ secondary educational level (minimum of 8 years) and 1445 (85.5%) were housewives. The mean (SD) of the age and parity were 27.2 (6.3) years and 2.0 (2.1) respectively. Out of these 1690 women, 94(5.5%) were underweight (BMI of ≤ 19.9 Kg/m2), 603 (35.6%) were overweight (BMI of 25 - 29.9 Kg/m2) and 328 (19.4%) were obese (BMI of ≥ 30 Kg/m2).

In multivariate analyses, obesity was positively associated with age (OR = 1.2, 95% CI = 1.0-1.1; *P*< 0.001), and with women's education (OR = 1.8, 95% CI = 1.2-2.7; *P *= 0.001). Obesity was positively associated with parity in univariate analyses only (OR = 1.1, 95% CI = 1.0-1.2; *P *= 0.02)

**Conclusion:**

The high prevalence of obesity in these pregnant women represents a competing public health problem in Sudan. More research is needed.

## Introduction

Many developing countries are currently affected by high rates of overweight that in some cases surpass underweight as a public health nutritional problem. Recent reports showed that, in many developing countries e.g. Bangladesh, Nepal, and India, the prevalence of overweight-obesity in women of reproductive age has risen steadily in the last two decades [1]. In the case of Africa, recent analyses of national data on body mass index (BMI) from women showed that, the prevalence of overweight-obesity exceeded that of underweight [2].

Increasing BMI is associated with increased incidence of pre-eclampsia, gestational hypertension, macrosomia, induction of labor and caesarean delivery [3]. We have recently observed that, both type of malnutrition (underweight and overweight-obesity) were associated with poor pregnancy outcomes in pregnant Sudanese women [4,5]. Generally few data are available concerning epidemiology of obesity during pregnancy in Sub-Saharan Africa and none available for Sudan the largest African country with 40 million populations. Sudan has traditionally been known as to have the highest rates of poverty -and may be underweight in women- worldwide and there has been limited research to investigate how the nutritional status of women has changed during the recent period of economic development following oil industry. Investigating obesity during pregnancy and labour is of paramount importance for care-givers, health planners as well as managing clinicians so as to have fundamental data necessary for intervention. Thus, the current study was conducted to investigate prevalence of underweight, overweight and obesity among term pregnant women in Khartoum teaching hospital, Sudan.

## Materials and methods

The study was conducted during the period of February-April 2008 at Khartoum teaching hospital, Sudan, to investigate prevalence and risk factors for underweight, overweight-obesity among term pregnant women. Khartoum hospital provides tertiary care for women who receive a free antenatal care at the hospital, as well as for referrals from other clinics and hospitals, and for women who live close to the hospital. All women with risk factors or obstetric complications are referred to this hospital. However, the referral criteria are not strictly adhered to and many patients without any significant complications deliver at the hospital. After an informed consent, women with a singleton baby have been approached to participate in the study. A structured questionnaire was administered to each woman to gather information on educational level, age, parity and last menstrual period. The date of the last normal menstrual period was used to determine the gestational age at delivery. However, when the discrepancy between the gestational age determined in this way and the gestational age calculated from ultrasound scanning was greater than 2 week, the ultrasound estimate was preferred. Maternal weight and height were measured and expressed as body mass index (BMI - weight (kg)/height (m) ^2^). BMI was classified as underweight: less than or equal to a BMI of 19.9 Kg/m2. Normal: BMI of 20 - 24.9 Kg/m^2^. Overweight: BMI of 25 - 29.9 Kg/m^2^. Obese: BMI of 30 - 34.9 Kg/m^2^. Morbidly obese: BMI ≥ 35 Kg/m^2 ^[6]. The group with BMI in the normal range (20 - 24.9 Kg/m^2^) was used as the reference or comparison group for the analysis.

### Statistic analysis

Data were entered into a computer database and SPSS software (SPSS Inc., Chicago, IL, USA) and double checked before analysis.

Means and proportions for the socio-demographic characteristics were compared between the four subgroups of the BMI (underweight, normal, overweight and obese) in study using student t-test, ANOVA and *X*^2 ^respectively.

Univariate and multivariate analyses were performed, maternal BMI was dichotomized according to the above mentioned subgroups, here obesity (versus those with normal BMI, 20-24.9) was the dependent variable and socio-demographic (age, parity, gestational age, occupation and education) characteristics were independent variables. *P *- value < 0.05 was considered significant. In case of inconsistency between the results of ANOVA, *X*^2 ^and the results of multivariate analyses, the later was taken as final.

### Ethics

The study received ethical clearance from the Research Board at the Faculty of Medicine, University of Khartoum.

## Results

Out of 1890, 1690 women had complete data, hence included in these final analyses. Out of these 1690 women, 628 (37.1%) were primigravidae, 926 (54.8%) had ≥ secondary education level (minimum of 8 years) and 1445(85.5%) were housewives. The mean (SD) of the age, parity and gestational age were 27.2 (6.3) years, 2.0 (2.1) and 38.1 (1.8) weeks, respectively.

Out of these 1690 women, 94(5.5%) were underweight, 665(39.3%) had normal weight, 603 (35.6%) were overweight and 328 (19.4%) were obese. Both age and parity were significantly higher in the obese women. Gestational age was not significantly different between these subgroups. Significantly high numbers of obese women had ≥ secondary education level. There was no significant difference in the number of housewives between the BMI subgroups, table 1.

**Table 1:** Socio-demographic characteristics of the term pregnant women in the different BMI subgroups. *

Variables	Underweight (*N *= 94)	Normal weight (*N *= 665)	Overweight *(N *= 603)	Obese (*N *= 328)	*P*
Age, years	24.7(5.8)	26.1(6.4)	27.9(6.0)	30.3 (16.0)	< 0.001
Parity	1(0-9)	1(0-13)	2.0(0-12)	2.0(0-12)	0.002
					
Primigravidae	37(39.4)	291(43.8)	209(34.6)	91(27.7)	< 0.001
Gestational age, weeks	38.3(1.9)	37.9 (2.5)	38.2(2.3)	38.1(1.8)	0.1
Women's education ≥ secondary level	41(43.7)	309(46.5)	363 (60.2)	213 (65.0)	< 0.001
Job, housewife	87(92.5)	588(88.4)	488(80.9)	282(85.9)	0.1

• Data were shown as mean (SD) or number (%) for all variables (as applicable) except for parity where it was shown as median (range).

### Predicators for obesity

Women job (housewives vs. employees) was not significantly associated with obesity in multivariate analyses. Obesity was positively associated with age, i.e those who had age ≥ 27.2 years vs. those of age < 27.2 years (OR = 1.2, 95% CI = 1.0-1.1; *P *< 0.001), and with women's education of ≥ secondary level vs. education < secondary level (OR = 1.8, 95% CI = 1.2-2.7; *P *= 0.001) in both univariate and multivariate analyses. Obesity was positively associated with parity in univariate analyses only (OR = 1.1, 95% CI = 1.0-1.2; *P *= 0.02), table 2.

**Table 2:** Factors associated with obesity among term pregnant women in Khartoum hospital, Sudan using univariate and multivariate analyses

Variable	Univariate analyses			Multivariate analyses		
	**OR**	**95% CI**	***P*-value**	**OR**	**95% CI**	***P*-value**

Age, ≥ 27.2 vs. < 27.2 years	1.3	1.0-1.3	0.1	1.2	1.0-1.1	< 0.001
Parity	1.1	1.0-1.2	0.02	0.7	0.5-1.0	0.1
Women's education ≥ secondary level vs. < secondary level	2.2	1.6-2.9	< 0.001	1.8	1.2-2.7	0.001
Job, housewife vs. Employees.	1.2	0.9-1.5	0.06	0.7	0.5-1.0	0.08

Abbreviations: OR, Odds Ratio; CI, confidence interval.

## Discussion

This is the first published data concerning the epidemiology of underweight overweight and obesity among term pregnant women in Sudan. The main findings of the study were; the high prevalence (19.4%) of obesity among these women and obesity was positively associated with age, and with women's higher education. Recent reports from Bangladesh, Nepal, and India showed that, the prevalence of overweight-obesity in women of reproductive age has risen between 1996 and 2006 and overweight-obesity was positively related to age [1]. In neighboring Tanzania, the prevalence of obesity among pregnant women rose steadily and progressively from 3.6% in 1995 to 9.1% in 2004, obesity was positively associated with age, parity, and socioeconomic status and inversely with HIV infection [7]. However, these data should be compared cautiously because of the methodological differences. In the later studies these were the national surveys data and antenatal care data while in ours, it is the hospital - based data among parturient women. Interestingly in Tanzanian data the influence of HIV on underweight was obvious. HIV infection has become more prevalent and must now be considered as a possible etiological factor for malnutrition among pregnant women in Sub-Saharan Africa. Yet, for ethical reasons we could not investigate HIV among these women. Previously we have shown that pregnant women in central Sudan had a low HIV prevalence and they were poor uptakes for HIV testing and counseling [8,9]. It is necessary to point to the limitation of using anthropometric measurements taken during pregnancy. Unlike measurements before pregnancy, these measurements are liable to changes; unfortunately pre-pregnancy measurements can seldom be taken in Africa, where women commonly present to health facilities only when they are advanced in pregnancy. In Brazil, weight change associated to reproduction was highly dependent on BMI before pregnancy [10].

Educational level was associated with higher prevalence of overweight-obesity in Bangladesh, Nepal, and India [1] as well as in this study. This is may be the result of shifts from manual labor to more sedentary occupations and the related decline in physical activity. Although, there is no published data on physical activity among Sudanese women, there is evidence that sedentary lifestyles are becoming more common among urban Indian women [11]. Furthermore, the cultural perception toward weight and body image may be different in the different setting; e.g. obesity could be perceived as a sign of prosperity as opposed to the stigma that exists in some developed societies [12].

In the current study, obese pregnant Sudanese women had higher parity and parity was associated with obesity in univariate analyses but this association disappeared in multivariate analyses. This indicated that parity was a confounder in this study. Recently parity was associated with increased waist circumference and other anthropometric indices of obesity in a sample of rural Iraqi women attending two primary health care centers [13]. Likewise, in neighboring Tanzania obesity was positively associated parity [7].

Recently Villamor and colleagues (2006) observed that obesity was positively associated with socio-economic status among Tanzanian pregnant women [7]. In chilly marital status, employment and smoking were associated with obesity [14]. Smoking and the use of alcohol and other substances are not common among Sudanese women and all women who presented in this study were married. These factors have not been investigated, because they are difficult to investigate and may be considered as confounders in our study. Sudan is the largest African country with 40 million inhabitants with various cultural and social backgrounds that make it difficult to investigate the socio-economic status among these pregnant women. Moreover body weight (specially obesity) could be predicted by many other important factors apart from those included in the study, like family history, some endocrinal disorders (Cushing and Hypothyroidism), and nutritional habits. For technical and fund constrains, It was difficult to investigate all of these factors in this work.

## Conclusion

In summery these are hospital- based data which may not represent what is going in the community. Women tend to present to the hospital when they have complications. The high prevalence of obesity in these pregnant women represents a competing public health problem in Sudan. More research is needed in the area of maternal and perinatal outcomes of obesity during in this setting.

## Competing interests

The authors declare that they have no competing interests.

## Authors' contributions

DER and IA carried out the study and participated in the statistical analysis and procedures. AOA, YS and TED coordinated and participated in the design of the study, statistical analysis and the drafting of the manuscript. All the authors read and approved the final version.

## References

[B1] Balarajan Y Villamor E Nationally representative surveys show recent increases in the prevalence of overweight and obesity among women of reproductive age in Bangladesh, Nepal, and India J Nutr 2009 139 2139 2144 10.3945/jn.109.112029 19776182

[B2] Mendez MA Monteiro CA Popkin BM Overweight exceeds underweight among women in most developing countries Am J Clin Nutr 2005 81 714 721 15755843 10.1093/ajcn/81.3.714

[B3] Bhattacharya S Campbell DM Liston WA Bhattacharya S Effect of Body Mass Index on pregnancy outcomes in nulliparous women delivering singleton babies BMC Public Health 2007 7 168 10.1186/1471-2458-7-168 17650297 PMC1940246

[B4] Adam I Babiker S Mohmmed AA Salih MM Prins MH Zaki ZM Low body mass index, anaemia and poor perinatal outcome in a rural hospital in eastern Sudan J Trop Pediatr 2008 54 202 204 10.1093/tropej/fmm110 18156642

[B5] Alhaj AM Radi EA Adam I Epidemiology of preterm birth in Omdurman Maternity hospital, Sudan J Matern Fetal Neonatal Med 2010 23 131 134 10.3109/14767050903067345 19565427

[B6] Abrams B Parker J Overweight and pregnancy complications Int J Obes 1988 12 293 303 3058615

[B7] Villamor E Msamanga G Urassa W Petraro P Spiegelman D Hunter DJ Fawzi W W Trends in obesity, underweight, and wasting among women attending prenatal clinics in urban Tanzania 1995-2004 Am J Clin Nutr 2006 83 1387 1394 16762951 10.1093/ajcn/83.6.1387

[B8] Gassmelseed DEA Nasr AM Homeida SM Elsheikh MA Adam I Prevalence of HIV infection among pregnant women of the central Sudan J Med Virol 2006 78 1269 1270 10.1002/jmv.20694 16927279

[B9] Mahmoud MM Nasr AM Gassmelseed DEA Abdalelhafiz MA Elsheikh MA Adam I Knowledge and attitude toward HIV voluntary counseling and testing services among pregnant women attending an antenatal clinic in Sudan J Med Virol 2007 79 469 473 10.1002/jmv.20850 17385672

[B10] Coitinho DC Sichieri R D'Aquino Benício MH Obesity and weight change related to parity and breast-feeding among parous women in Brazil Public Health Nutr 2001 4 865 870 10.1079/PHN2001125 11527509

[B11] Singh RB Pella D Mechirova V Kartikey K Demeester F Tomar RS Beegom R Mehta AS Gupta SB De Amit K Neki NS Haque M Nayse J Singh S Thakur AS Rastogi SS Singh K Krishna A Prevalence of obesity, physical inactivity and undernutrition, a triple burden of diseases during transition in a developing economy. The Five City Study Group Acta Cardiol 2007 62 119 127 10.2143/AC.62.2.2020231 17536599

[B12] Prentice AM The emerging epidemic of obesity in developing countries Int J Epidemiol 2006 35 93 99 10.1093/ije/dyi272 16326822

[B13] Mansour AA Ajeel NA Parity is associated with increased waist circumference and other anthropometric indices of obesity Eat Weight Disord 2009 14 50 55 10.1007/BF03327800 19934637

[B14] Koch E Bogado M Araya F Romero T Díaz C Manriquez L Paredes M Román C Taylor A Kirschbaum A Impact of parity on anthropometric measures of obesity controlling by multiple confounders: a cross-sectional study in Chilean women J Epidemiol Community Health 2008 62 461 470 10.1136/jech.2007.062240 18413461

